# Covid-19 Quarantine: Impact of Lifestyle Behaviors Changes on Endothelial Function and Possible Protective Effect of Beetroot Juice

**DOI:** 10.3389/fnut.2020.582210

**Published:** 2020-10-21

**Authors:** Mônica Volino-Souza, Gustavo Vieira de Oliveira, Carlos Adam Conte-Junior, Thiago Silveira Alvares

**Affiliations:** ^1^Nutrition and Exercise Metabolism Research Group, Nutrition Institute, Federal University of Rio de Janeiro, Rio de Janeiro, Brazil; ^2^Graduate Program in Food Science (PPGCAL), Institute of Chemistry (IQ), Federal University of Rio de Janeiro (UFRJ), Cidade Universitária, Rio de Janeiro, Brazil; ^3^Nanotechnology Network, Carlos Chagas Filho Research Support Foundation of the State of Rio de Janeiro (FAPERJ), Rio de Janeiro, Brazil; ^4^Postgraduate Program in Bioactive Products and Biosciences, Federal University of Rio de Janeiro, Rio de Janeiro, Brazil

**Keywords:** dietary nitrate, nitric oxide, vascular health, cardiovascular disease, coronavirus

## Abstract

The current recommendation for reducing person-to-person Coronavirus 2019 (COVID-19) transmission is social distancing, including remote work and school, and home confinement. However, confinement may cause negative feelings, such as frustration, anger, boredom, and stress, in quarantined people. Furthermore, unhealthy diet and physical inactivity behaviors are commonly linked to home confinement, leading to weight gain, metabolic disorders, smoking, and exacerbated alcohol consumption. As a result, these unhealthy behaviors are typically linked to vascular endothelium damage (endothelial dysfunction), which is a first step for the development of cardiovascular disease (CVD). Given that CVD is the main cause of morbidity and mortality worldwide, attenuating the progression of endothelial dysfunction is very important for the control of CVD. Consuming vegetable rich in nitrate, such as beetroots, may be an effective way to prevent endothelial dysfunction. Several emerging studies have recommended beetroot juice in order to improve endothelial function in hypertensive, hypercholesterolemic individuals, as well as in those with CVD risk factors. Therefore, nitrate-rich vegetable consumption, such as beetroot, should be encouraged to be included in the diet during confinement from COVID-19 outbreaks in order to alleviate the potential negative effect of home confinement on cardiovascular health.

## Introduction

Coronavirus disease 2019 (COVID-19) emerged at the end of 2019 in Wuhan, China. It manifests as either an asymptomatic infection or mild to severe pneumonia. In March 2020, COVID-19 was declared a pandemic by the World Health Organization (WHO), and since several reports have suggested that person-to-person transmission is the most likely route for COVID-19 contamination, quarantine has been widely adopted (1).

Although quarantine is very important in controlling of COVID-19 person-to-person transmission, several studies have been demonstrating that quarantine promotes changes in lifestyle behavior that can negatively impact the cardiovascular health (2, 3). Social distancing and negative feelings (i.e., frustration, boredom, financial loss and stigma) during quarantine have been associated with chronic stress, systemic inflammation, and oxidative stress (4). Furthermore, physical activity is reduced while the ingestion of high-caloric foods is increased (2).

Taken together, these factors may contribute to endothelial dysfunction by reducing nitric oxide (NO) bioavailability. Changes in NO bioavailability are known to play a role in the development of a number of clinical conditions in which the function of the vascular system is impaired. Evidence indicates that the most likely mechanism for endothelial dysfunction is that of a reduced bioavailability of NO as a result of its interaction with reactive oxygen species (ROS), specifically superoxide anion (5). The inactivation of NO by superoxide anion is an example of what is called oxidative stress, a term used to describe an imbalance between the anti-oxidant defenses of cells and excessive formation of ROS (5). Since the adoption of new unhealthy life habits due to confinement (i.e., drinking, smoking, unbalanced diet, stress, and sedentary life) may contribute to exaggerated production of reactive oxygen species (ROS), people under quarantine may be more susceptible to increase their risk for developing cardiovascular disorders.

It is recognized that the ingestion of vegetables and fruits can improve the endothelial function due to bioactive compounds in its composition. Beetroot is an important vegetable source of nitrate, a bioactive compound that has widely investigated due to its positive effects on endothelial function (6, 7). Beetroot contains higher levels of nitrate that can be converted into nitrite by bacteria present in the oral cavity (8). Nitrite, in turn, can be converted into NO in dysfunctional arteries, improving endothelial function (6, 7). The present manuscript discusses how some unhealthy habits caused by COVID-19 quarantine may negatively affect endothelial function, which may be mitigated by beetroot juice ingestion to improve endothelial dysfunction. Additionally, we provide insight to encourage the consumption of dietary sources rich in nitrate (i.e., beetroot) for patients with COVID-19 who may have impaired NO bioavailability due to COVID-19 virus in endothelial cells, as well as present endothelial inflammation.

## Lifestyle Behavior Changes During Quarantine

Due to the COVID-19 pandemic, the main, worldwide recommendation is to restrict daily living, which includes remote work and school, social distancing, and home confinement. However, these restrictions (quarantine and social isolation) may negatively affect people's health, since home confinement reflects changes in lifestyle behaviors, including reduction of physical activity and overeating of unhealthy foods (2), which collectively may lead to metabolic disorders (i.e., overweight/obesity, diabetes, hypertension). In addition, social distancing caused by home confinement increases the risk of psychosocial strain (2, 3). A previous study has investigated the lifestyle behaviors of 1047 individuals from different countries during quarantine. It was reported that a greater proportion of individuals are experiencing psychosocial and emotional disorders, which were associated with unhealthy lifestyle behaviors, such as physical and social inactivity, unhealthy diet, and poor sleep quality (2).

**Figure 1:**
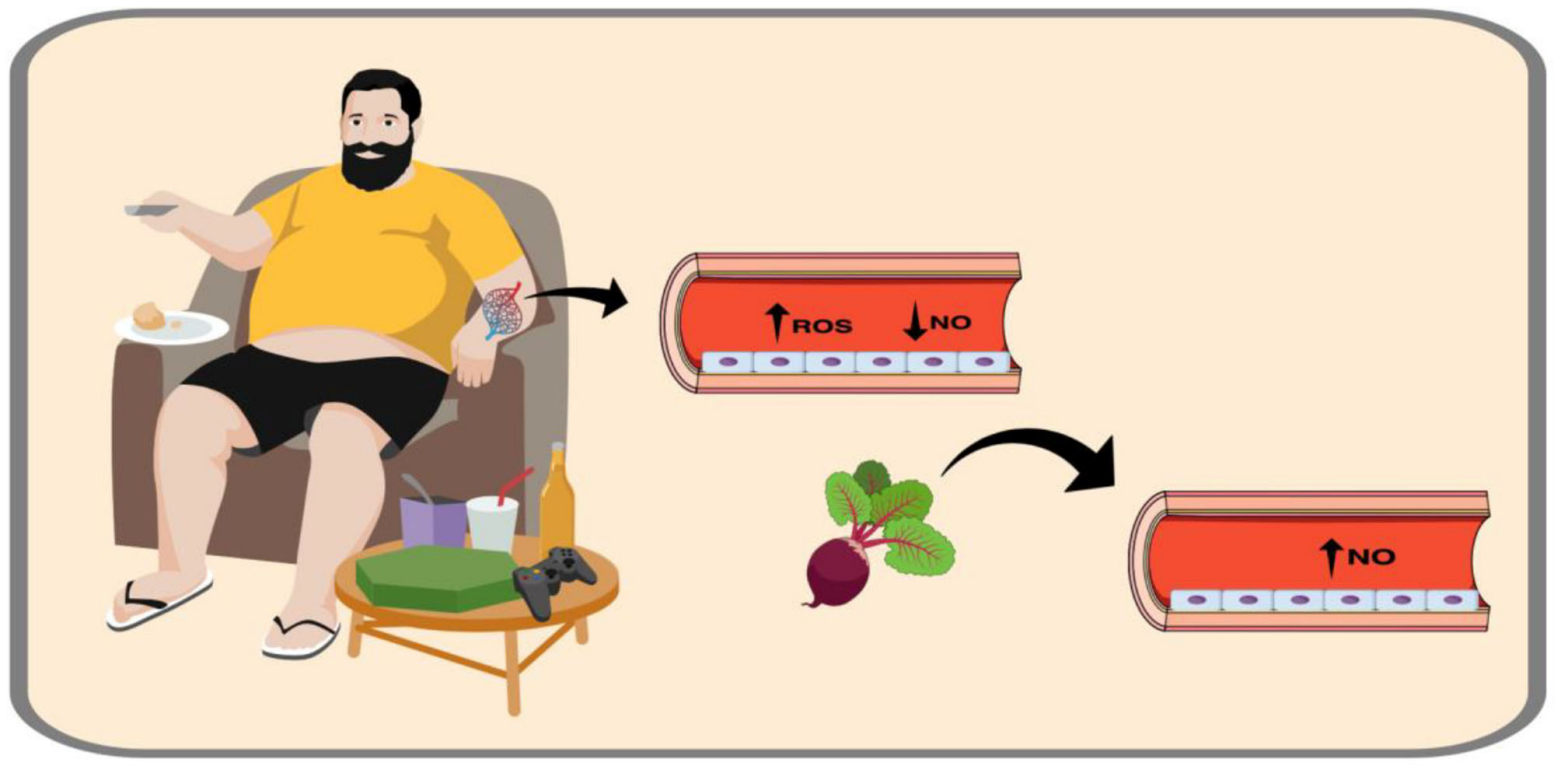
The effect of lifestyle behaviors changes during COVID-19 quarantine on endothelial function. Social distancing was adopted as a way to reduce person-to-person Coronavirus 2019 transmission. However, home confinement may promote lifestyle behaviors changes as unhealthy diet and physical inactivity. Collectively, these unhealthy behaviors are associated with endothelial damage, the first step for the development of cardiovascular diseases. Since nitrate-rich beetroot juice ingestion has demonstrated positive effect on endothelial function due to its effects on nitric oxide bioavailability, its ingestion could be encouraged during COVID-19 quarantine in order to alleviate the potential negative effect of home confinement on cardiovascular health.

Emotional disorders (i.e., depression, anxiety, frustration, boredom) have been linked to greater energy ingestion, mainly due to the intake of foods high in fat and sugar (9). Typically, individuals reach for unhealthy food (snacks) during stressful periods as a way to relieve tension caused by negative feeling, such as fear, confusion, stress, and anger during quarantine (3, 9). A diet high in fat and sugar can inhibit the satiety response in the brain, activating reward systems, and consequently leading to more food ingestion (10). Greater energy intake combined with decreased physical activity levels due to confinement may result in weight gain, as well as elevated blood pressure, glucose, and lipids, thereby increasing the risk for cardiovascular disease (CVD) (Figure 1). Moreover, it is important to note that CVD is one of the main causes of mortality and motility worldwide; therefore, a possible means of attenuating and/or alleviating an eventual CVD progression during COVID-19 pandemic is needed.

## Impact of COVID-19 Quarantine on Vascular Health

Cardiovascular disease is mainly caused by endothelial dysfunction, an abnormal condition in which the endothelium cells (the inner layer of the arteries) fails to synthesize and release its vasoactive substances, such as NO (8). Nitric oxide is a gasotransmitter molecule synthesized in endothelial cells, which acts in smooth muscle cells surrogating the arteries, causing arteries relaxation (8). Therefore, when the NO bioavailability is negatively affected by some abnormal condition (oxidative stress and inflammation), vascular tonus regulation becomes dysfunctional, increasing the risk of a cardiovascular event.

In this context, endothelial dysfunction may occur during quarantine as a result of a combination of multiple factors (11). For example, the negative feelings caused by COVID-19 home confinement may incite unhealthy behaviors, including smoking, excessive alcohol consumption, and overeating high fat and sugar foods (12). Torres et al. (12) demonstrated that emotionally stressed individuals ingested high-fat food in order to feel better. Moreover, since most people are fearful of being infected with COVID-19, it is plausible that people will prefer to purchase packaged and longer shelf-life foods instead of vegetable and fruits (fresh food) in order to avoid frequent supermarket shopping. In the short- and long-term, such unhealthy food ingestion and reduced levels of physical activity are very likely to cause weight gain, increased blood glucose levels, systemic inflammation, oxidative stress, and blood pressure in people suffering from emotional disorders (11).

Inflammation and oxidative stress play a critical role in the progression of endothelial dysfunction (13). Excessive gain of body fat, mainly intra-abdominal fat depot (visceral obesity), insulin resistance, smoking, stress, and dyslipidemia are often linked to abnormal production of reactive oxygen species (ROS) (oxidative stress) and inflammatory response (14), contributing to the progression of atherosclerosis and endothelial dysfunction. In its simplest form, vascular inflammation induces exacerbated production of ROS, which interacts with NO molecules to form reactive nitrogen species (i.e., peroxynitrite), thereby reducing the bioavailability of NO (13). Furthermore, reactive nitrogen species and ROS can uncouple endothelial nitric oxide synthase enzyme (eNOS) via reductions in tetrahydrobiopterin, an important cofactor for the eNOS enzyme (15). In this scenario, the eNOS enzyme produces ROS in the place of NO, largely affecting NO bioavailability, which further contributes to the endothelial dysfunction.

Additionally, besides the fact that quarantined people may not be engaged in regular physical exercise, remote school/work may increase the time spent sitting at home (16). There is evidence showing that spending 3 h sitting can impair vascular function assessed in the popliteal artery (the main artery that feeds the leg structures) (17). Prolonged sitting reduces the blood perfusion in the lower extremities, as well as the venous returns, leading to a decline in cardiac output, and a subsequent reduction in leg blood flow-induced shear stress (17). Reduced blood flow-induced shear stress is associated with decreasing NO bioavailability, vascular dysfunction, and arterial stiffness (17). Moreover, it is apparent that quarantined individuals reduce physical activity because most people are currently staying at home. Boyle et al. (18) demonstrated that individuals who were experimentally undergone to diminish their daily physical activity (from <10,000 steps/day to <5,000 steps/days) during 5 days exhibited reduced endothelial function.

Taken together, many factors, including overeating and physical inactivity, may impair endothelial function of individuals experiencing home confinement during COVID-19 outbreaks, increasing the chance of a cardiovascular event.

## The Nitrate-Nitrite-Nitric Oxide Pathway

Nitric oxide can be produced by two different ways: one from the enzymatic L-arginine/NO synthase pathway, and another from a non-enzymatic nitrate-nitrate-NO pathway. To produce NO by L-arginine/NO synthase pathway, oxygen and L-arginine are substrates for eNOS enzyme, generating NO and L-citrulline (8). However, it has been demonstrated that some pathophysiological conditions (i.e., aging, hypertension, diabetes) may decrease the bioavailability of substrates for eNOS, as well as its functionality, impairing the NO production (15).

**Figure 2:**
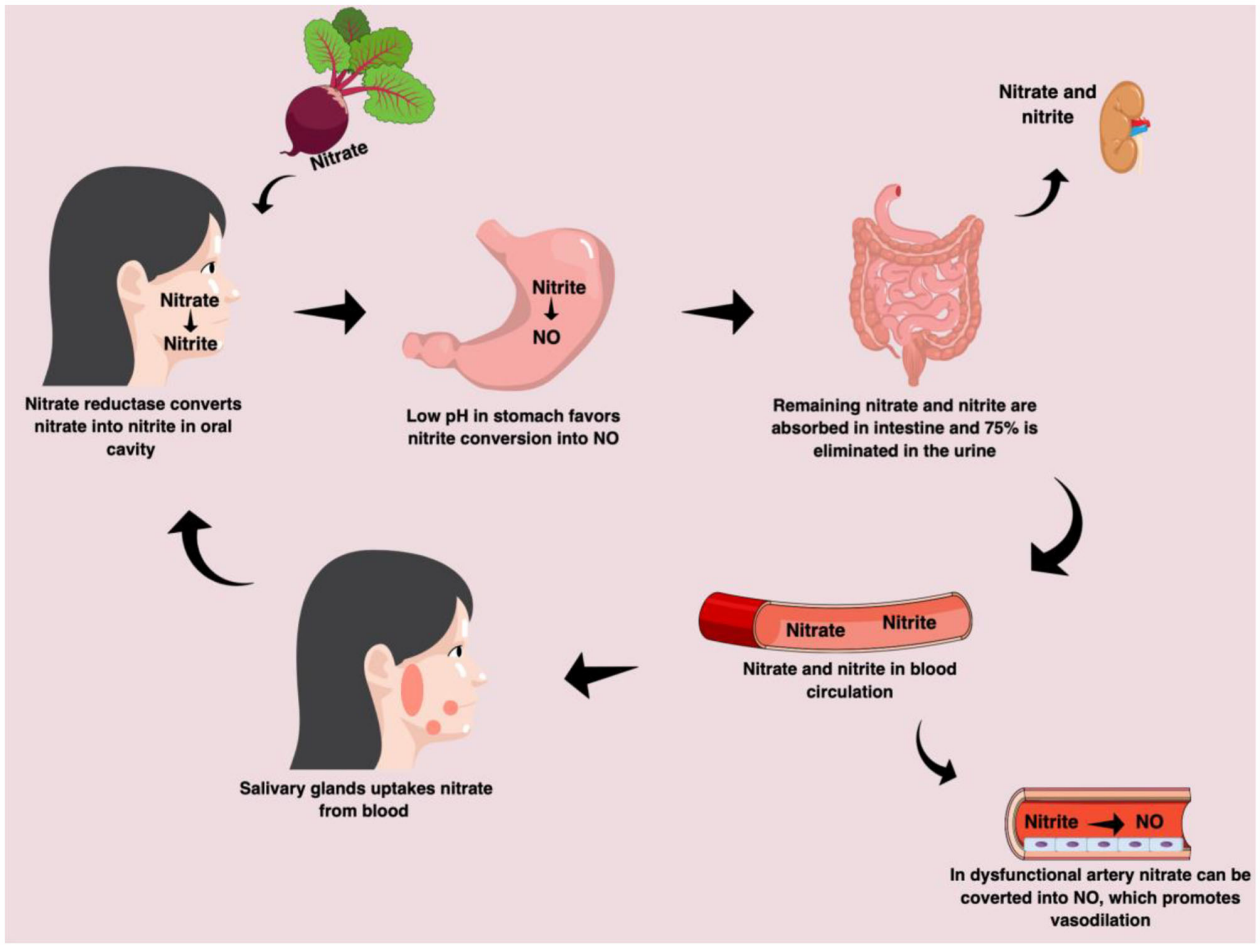
Nitrate-nitrite-nitric oxide pathway in humans. After ingestion of nitrate-rich beetroot juice, the nitrate is converted into nitrite by nitrate reductase enzymes in the oral cavity. In the stomach, due to low pH the nitrite can be converted into nitric oxide. The remaining nitrate and nitrite are absorbed from the intestine and 75% is excreted in the urine. The nitrite absorbed from intestine into blood circulation can be converted into nitric oxide in dysfunctional artery.

In contrast to the L-arginine/NO synthase pathway, the nitrate-nitrite-NO pathway is not dependent of eNOS enzyme, oxygen and L-arginine, thus the nitrate-nitrite-NO may be considered an alternative pathway for NO production (8). After ingestion of rich-nitrate foods, nitrate is converted to nitrite in oral cavity by commensal bacterial and then it is converted to NO in the stomach due to a favorable environment (i.e., low oxygen pressure and low pH) (8). Nitrate and remaining nitrite are absorbed in the intestine and an uptake of nitrate from the blood occur by salivary glands, a process called entero-salivary circulation. Thus, nitrate can promote increasing NO production when the L-arginine/NO synthase pathway is not efficient (8) (Figure 2).

Nitrate can mainly be found in food sources, such as green leafy vegetables (spinach and arugula), in spite of being also present in water and processed meats. Furthermore, nitrate can be found in roots, such as beetroot. For this reason, the effect of beetroot ingestion has been investigated in several clinical populations, such as hypertensive (6) and hypercholesterolemic (19) individuals, as a way to increase NO bioavailability and thus improve endothelial function.

## Can Beetroot Juice Ingestion Have an Impact on COVID-19 Quarantine-Induced Vascular Damage?

Beneficial effects of beetroot juice intake on endothelial function have been demonstrated in subjects with the presence of risk factors for cardiovascular disease (7, 19) given the nitrate present in beetroot can be reduced to nitrite in oral cavity, and then nitrite to NO in dysfunctional arteries. Although nitrate itself has been proposed to present some biological effect, its action on endothelial function seems particularly to be related to the nitrate ability to generate nitrite-NO. For example, previous studies have demonstrated an improvement in flow-mediated dilation (FMD) response, a NO-mediated endothelium-dependent measurement for assessing endothelial function, of elderly people (7), patients with hypercholesterolemia (19) and hypertension (6) after acute or chronic beetroot juice consumption. The authors have attributed the positive effects of dietary nitrate on endothelial function by the increased NO bioavailability, which promotes vasodilation. The hypothesis that NO, instead nitrate itself, is involved in improved endothelial function may be reinforced through previous evidence that used FMD measurement as a tool for assessing endothelial function (20, 21).

As previously mentioned, the quarantine has promoted changes in lifestyle, which include increased stress and, consequently, increased ingestion of highly caloric foods. A positive effect of beetroot intake on vascular function after ingestion of high-calorie foods has been found. For example, Joris et al. (22) observed that ingesting 140 mL of beetroot juice attenuates the postprandial impairment of flow-mediated dilation (FMD) (a measure of endothelial function) following a meal composed of two muffins (containing 56.6 g of lipid). These findings are important since increased ingestion of high-calorie foods may occur during quarantine (3), which damages on vascular function (22). Therefore, these findings support the idea that beetroot juice may be an alternative way to attenuate the endothelial dysfunction induced by high-caloric ingestion during quarantine.

It has also been observed that damage to endothelial function can lead to hypertension (23), due to the reduction in vasoactive molecules, such as NO. Thus, given the potential effect of beetroot juice on increasing NO metabolites (nitrate and nitrite) in biological fluids (19), previous studies have demonstrated a reduction of systolic and diastolic blood pressure of normotensive (24), hypertensive individuals (25), and older adults (26).

Furthermore, it is plausible that changes in lifestyle behaviors during quarantine can also lead to an alteration in biochemical parameters. Previous studies have demonstrated that beetroot juice induced reductions in low density lipoprotein-cholesterol (LDL-C) and total cholesterol of hypertensive patients (25), and increases in high lipoprotein-cholesterol (HDL-C) of healthy individuals (27). Furthermore, beetroot juice induced a reduction in blood glucose in healthy people (28). Nitric oxide plays underlying role in lipids and glucose metabolism (29), which may explain the positive effect of beetroot juice on lipids parameters.

It is important to note that people may choose not to purchase fresh food because it involves more frequent shopping and consequently increase the probability of COVID-19 infection. Thus, the reduced consumption of vegetables and fruits (including beetroots), which are important source of bioactive compounds, such as nitrate may result in reduced ingestion of these compounds that are essential for endothelial function.

To mitigate this potentially unhealthy consumer trend during quarantine, there are storage strategies that can increase the vegetable/fruits shelf life. For instance, storage of foods under low temperatures may preserve bioactive compounds in vegetables and fruits (30). Corleto et al. (30) showed that nitrate content in beetroot juice was stable for 4 and 30 days when storage at 4 and −20°C, respectively. Therefore, beetroot juice may be beneficially consumed when stored under appropriate conditions (low temperature), preserving the nitrate content in beetroot juice for a longer period. Corleto et al. (30) showed that nitrate degradation from beetroot juice started within 24 h at 25°C and after 4 days at 4°C, while it was stable for 1 month at temperatures below −20°C. Therefore, when making the juice from beetroot it should be consumed immediately or if storage it should be at a temperature below −20°C (e.g., fridge freezer) for up to 1 month to avoid nitrate losses. Avoiding nitrate losses from beetroot juice is important since the beneficial cardiovascular effect of beetroot juice consumption is related to the presence of nitrate in this food.

## Can Beetroot Ingestion be Useful for COVID-19 Patients?

Coronavirus disease reflects a global outbreak of respiratory illness caused by the severe acute respiratory syndrome coronavirus-2 (SARS-CoV-2). Since no registered treatment or vaccine for COVID-19 has demonstrated to be effective, many general treatments, from nutritional interventions to antivirals utilized for others disease, have been extensively reviewed and proposed in order to alleviate and/or prevent the rapid progression to the respiratory illness and fulminant systemic organ failure caused by SARS-CoV-2 (31). The SARS-CoV-2 infection is accompanied by an aggressive inflammatory response with the release of a large amount of pro-inflammatory cytokines, which is an event known as “cytokine storm” (32). Previous studies investigating cytokines profile from COVID-19 patients indicated that “cytokines storm” is correlated with respiratory illness, multiple organ failure, and unfavorable prognosis of severe COVID-19 (33–36).

Inflammatory and immune response in COVID-19 can strongly affect the NO bioavailability since inflammation is associated with overproduction of ROS, which interacts with NO to generate peroxynitrite, a reactive nitrogen specie (RNS). Exacerbated production of ROS and RNS serve as host defense and are induced during stress, such as viral infection (35). Considering that SARS-CoV-2 infects endothelial cells, which are the major source of NO synthesis, change in normal NO/ROS balance in such cells can negatively affect the endothelial function (i.e., NO is a potent vasoactive molecule), as well as regulating inflammatory cascades (i.e., NO inhibits viral replication, reducing inflammatory response) (37). In this context, scientific evidence has demonstrated NO supplementation (by using inhaled NO or donor drugs, such as S-nitrosothiols) under pro-inflammatory conditions may prevent cytokine storm and restores microvascular function (37, 38).

It has also been suggested that natural products that boost NO production may be possibilities in treating the COVID-19 (38). In this sense, beetroot juice (a natural product) could be an interesting option to delivery nitrate, a nutrient that can be reduced to nitrite in the body and then increase NO bioavailability in COVID-19 patients. Although no clinical trials testing natural products has yet been conducted, it would be plausible to investigate the effect of beetroot in COVID-19 patients given the effectiveness of beetroot juice ingestion in increasing NO precursors (nitrate and nitrite) in plasma and urine (39, 40). A previous study has suggested curcumin as a possible natural product that can be explored for early intervention in COVID-19 due to the anti-inflammatory effect of curcumin (38). However, it should be note that possible interactions between natural products and medication currently utilized for treating COVID-19 must be taken into consideration.

## Conclusion

In summary, the home confinement caused by COVID-19 outbreaks may lead to stress, physical inactivity, and increased high-calorie food consumption. Taken together, these factors may impair endothelial function, which is the first step for the development of CVD. Beetroot juice ingestion has been largely encouraged due to its positive effect on endothelial function in several populations and it may be part of a strategy to alleviate the negative effect of home confinement during COVID-19 outbreaks. Additionally, futures studies could investigate the effect of beetroot on COVID-19 patients, given the potential impact of this vegetable in increasing NO precursors.

## Data Availability Statement

The original contributions generated in the study are included in the article/supplementary material, further inquiries can be directed to the corresponding author/s.

## Author Contributions

MV-S and GO were responsible for writing the manuscript. TA and CC-J contributed reviewing the manuscript. All authors contributed to the article and approved the submitted version.

## Conflict of Interest

The authors declare that the research was conducted in the absence of any commercial or financial relationships that could be construed as a potential conflict of interest.
